# Bromelain capped gold nanoparticles as the novel drug delivery carriers to aggrandize effect of the antibiotic levofloxacin

**DOI:** 10.17179/excli2016-710

**Published:** 2016-12-06

**Authors:** Paramdeep Bagga, Tarique Mahmood Ansari, Hefazat Hussain Siddiqui, Asad Syed, Ali H. Bahkali, Md. Azizur Rahman, Mohd. Sajid Khan

**Affiliations:** 1 Department of Pharmacy, Integral University, Lucknow, Uttar Pradesh 226026 (India); 2 Botany and Microbiology Department, Faculty of Science, King Saud University, Riyadh, Saudi Arabia; 3 Nanotechnology Lab, Department of Biosciences, Integral University, Lucknow, Uttar Pradesh 226026 (India)

**Keywords:** antibacterial activity, bromelain, gold nanoparticles, levofloxacin, novel drug delivery carriers

## Abstract

To develop bromelain capped gold nanoparticles (BRN capped Au-NPs) as the effective drug delivery carriers of the antibiotic levofloxacin (LvN) and evaluate antibacterial potential of its bioconjugated form compared to pure LvN. BRN capped Au-NPs were synthesized by *in vitro* method and bioconjugated to LvN using 1-ethyl-3-(3-dimethylamino-propyl)-carbodiimide as activator to form Au-BRN-LvN-NPs. These were characterized for mean particle size by dynamic light scattering analysis, zeta potential by Zetasizer nanosystem analysis and transmission electron microscopy (TEM) on carbon coated TEM copper grids by TEM respectively. Drug loading efficiency of LvN was calculated using UV-visible spectroscopy by standard curve of pure LvN. Antibacterial efficacy of Au-BRN-LvN-NPs and pure LvN was determined by evaluating minimum inhibitory concentration (MIC) against *Staphylococcus aureus* and *Eschereschia coli*. Two peaks were observed in Au-BRN-LvN-NPs spectrum one at 307 nm and other at 526 nm while one peak in BRN capped Au-NPs at 522 nm during UV spectroscopy suggesting red shift. The drug loading efficiency of LvN was found to be 84.8 ± 2.41 %. The diameter of Au-BRN-LvN-NPs and BRN capped Au-NPs were found to be (58.65 ± 2 nm, 38.11 ± 2 nm), zeta potential (-9.01 mV, -13.8 mV) and surface morphology (~13.2 nm, 11.4 nm) respectively. The MICs against *S. aureus* and *E. coli* were found to be (0.128 µg/mL, 1.10 µg/mL) for Au-BRN-LvN-NPs and (0.547 µg/mL, 1.96 µg/mL) for pure LvN. The results suggested that BRN capped Au-NPs can be used as effective drug delivery carriers of the antibiotic LvN. The Au-BRN-LvN-NPs exhibited enhanced antibacterial activity compared to pure LvN alone. (Graphical abstract see Figure 1(Fig. 1))

## Introduction

The metallic nanoparticles derived from noble metals such as gold has attracted the researchers all over the world regarding their escalating use in delivery of drugs (Emerich and Thanos, 2006[10]). These particles build up new physico-chemical properties which are absent in individual molecules. Specially, the gold nanoparticles are being targeted nowadays by the researchers since it not only encompasses the advantages of “nano-sizing” but it also includes surface functionalization necessary to augment stability and enable conjugation with biomolecules (Couto et al., 2016[7]). Surface functionalization of the gold nanoparticles has opened new frontiers for delivery of drugs such as anticancer and the antimicrobial drugs (Dhar et al., 2008[8], Selvaraj and Alagar, 2007[24]). Bovine serum albumin capped gold nanoparticles of amino-glycosidic antibiotics and the acridine derivatives bioconjugated on citrate stabilized gold nanoparticles have been used as the effective drug delivery vehicles (Rastogi et al., 2012[22], Mitra et al, 2014[18]). Thus, these exciting approaches have motivated the search for novel molecules for the synthesis of gold nanoparticles.

Levofloxacin (LvN) which is chemically (*S*)-9-fluoro-2,3-dihydro-3-methyl-10-(4-methylpiperazin-1-yl)-7-oxo-7H-pyrido[1,2,3-de]-1,4-benzoxazine-6-carboxylic acid, is a broad spectrum fluoroquinolone antibiotic active against both Gram positive and Gram negative bacteria functioning by inhibiting the type II topoisomerase enzymes namely DNA gyrase and topoisomerase IV (Drlica and Zhao, 1997[9]). It is recommended as a first-line treatment option by 'Infectious Disease Society of America' for catheter associated urinary tract infections in adults and in prosthetic joint infection in combination with rifampicin (Hooton et al., 2010[12]; Osmon et al., 2013[20]).

Bromelain (BRN) is a proteolytic enzyme derived from the plant *Ananas comosus* belonging to family Bromeliaceae (Taussig and Batkin, 1988[26]). It has shown analgesic, anti-inflammatory, anti-arthritis, antitumuor, anthelminthic, antimicrobial and several other pharmacological activities (Leipner et al, 2001[16]; Beez et al, 2007[3]; Rowan et al., 1990[23]; Praveen et al., 2014[21]). It was found to be less toxic having mutagenic potential (Castell et al., 1997[5]; Chobotova et al., 2010[6]). Hence, the present study was aimed to develop bromelain capped gold nanoparticles as the effective drug delivery carrier of the antibiotic levofloxacin and to evaluate the antibacterial potential of its bioconjugated form as compared to pure LvN alone.

## Materials and Methods

### Chemicals and reagents

All the chemicals and solvents used in the present investigation were of analytical grade. Levofloxacin was procured from Yarrow Chem Products, Mumbai (India) and bromelain from Merck Chemicals Darmstadt, Germany.

### Synthesis of gold nanoparticles

Gold nanoparticles (Au-NPs) were synthesized by *in vitro* method. The 3 μl of 1.0 mM H[AuCl_4_] prepared in 50 mM phosphate buffer was taken in 3 mL of freshly prepared bromelain (0.33 mg/mL) and incubated at the temperature of 40 °C for 48 h in an incubator. The reaction mixture without bromelain was used as a control. To ensure the formation of nanoparticles, the samples were removed from the reaction mixture at regular intervals and analyzed by UV-visible spectroscopy. On completion of the reaction, Au-NPs were collected by centrifugation at 30000 g for 30 min, washed twice with MilliQ water and the excess bromelain was removed by treating with 50 % ethanol (v/v) (Khan et al., 2015[15]).

### Bioconjugation of gold nanoparticles to levofloxacin

Synthesized Au-NPs were bioconjugated to LvN by using 1-ethyl-3-(3-dimethylamino-propyl)-carbodiimide (EDC) as the activator (Timkovich, 1977[27]). The 5 mM EDC was added to the 5 ml reaction mixture containing 250 µg of LvN, 250 µg of Au-NPs and 50 mM MES/HEPES buffer (HiMedia laboratories, India) in aliquots within 3 h at 30 °C for the coupling process. The bioconjugates so formed were separated from unconjugated Au-NPs by passing the reaction mixture through Biogel P-30 gel filtration column pre-equilibrated with 20 mM HEPES buffer (*pH* 6.0) containing 150 mM sodium chloride. The fractions were scanned between 200-900 nm and subsequently pooled. The pooled samples were then dialyzed against distilled water and used for further characterization (Khan et al., 2015[14]). The samples were removed at regular intervals and analyzed in UV-visible spectroscopy (Shimadzu dual-beam spectrophotometer, model UV-1601 PC, Japan) at a resolution of 1 nm to confirm the formation of 'BRN capped Au-NPs' and 'levofloxacin conjugated with BRN capped Au-NPs'(Au-BRN-LvN-NPs).

### Characterization of synthesized gold nanoparticles and bioconjugated gold nanoparticles

#### Measurement of mean particle size and zeta potential of 'BRN capped Au-NPs' and Au-BRN-LvN-NPs

The samples of 'BRN capped Au-NPs' and Au-BRN-LvN-NPs were sonicated separately for 1 min and then, taken in a DTS0112-low volume disposable sizing cuvette of 1.5 mL for the measurement of mean particle size with a dynamic light scattering (DLS) particle size analyzer (Zetasizer Nano-ZS, model ZEN3600, Malvern Instrument Ltd, Malvern, UK) (Akhtar et al., 2014[1]). Zeta potential values were also assessed to check the stability of the gold nanoparticles with a Malvern Zetasizer Nanosystem (Worcestershire, UK).

#### Transmission electron microscopy of 'BRN capped Au-NPs' and Au-BRN-LvN-NPs

Transmission electron microscopy (TEM) was analyzed by drop drying the solution of biosynthesized 'BRN capped Au-NPs' and Au-BRN-LvN-NPs on carbon coated TEM copper grids followed by measurements on TEM (FEI Company, Tecnaiä G2 Spirit BioTWIN) at an accelerating voltage of 80 kV.

#### Fourier transform infrared spectroscopy of 'BRN capped Au-NPs' and Au-BRN-LvN-NPs

The Fourier transform infrared (FTIR) spectra of 'BRN capped Au-NPs' and Au-BRN-LvN-NPs were recorded using Perkin-Elmer Spectrum Two FT-IR (Perkin Elmer Inc., Tres Cantos, Madrid) equipped with a Universal attenuated total reflectance sampling device and scanned at room temperature in transmission mode over the wave number range of 4000-650 cm^-1 ^at a resolution of 4 cm^-1^.

#### Drug loading efficiency of levofloxacin

Drug loading efficiency of LvN was calculated using UV-visible spectroscopy at the wavelength of 307 nm/526 nm where the standard curve of the pure drug LvN was established and the amount of unbound LvN was calculated from it. The actual amount of bioconjugated LvN was calculated by subtracting unbound LvN from the total amount of LvN added. The amount of bioconjugated LvN was calculated using the equation [ % bioconjugation = (amount of drug conjugated / total drug added) × 100].

### Evaluation of antibacterial efficacy of bioconjugated levofloxacin, Au-BRN-LvN-NPs over pure levofloxacin alone

The antibacterial efficacy of bioconjugated LvN (Au-BRN-LvN-NPs) was determined by evaluating the minimum inhibitory concentration (MIC) of Au-BRN-LvN-NPs and pure LvN against Gram positive bacteria *Staphylococcus aureus* (NCIM No. 2079) and Gram negative bacteria *Eschereschia coli* (NCIM No. 2065) (Amsterdam, 1996[2]). The bacterial strains in mid logarithmic phase were harvested by centrifugation, washed with 10 mM sodium phosphate buffer (PB) of *pH* 7.4, and diluted to 2×105 colony forming units per ml (CFU/mL) in PB containing 0.03 % Luria-Bertani (LB) broth. Au-BRN-LvN-NPs were serially diluted in 50 µL of LB medium in 96-well microtitre plates so as to achieve the desired concentrations with bacterial inoculums (5×104 CFU/well) and were incubated at 37 °C overnight. The MIC was taken as the lowest Au-BRN-LvN-NPs concentration at which growth was inhibited (Khan et al., 2008[13]). For agar plate count method (Stenger et al., 1998[25]), 25 µL aliquot of bacteria (1×105 CFU/mL) was incubated with 25 µL of samples at 37 °C for 2 h (Hamamoto et al., 2002[11]). The mixtures were 10-fold serially diluted in PB, plated on LB agar and then incubated overnight at 37 °C. Bacterial colonies were enumerated following the day. After the determination of the MIC from the microtitre plate wells with no visible growth, the samples were removed for serial subcultivation (2 µL) into microtitre plates containing 100 µL of broth/well and further incubated for 24 h in order to determine the bactericidal activity measurement (MBC), the lowest concentration at which there is no visible bacterial growth indicating 99.5 % killing of the original inoculums. The absorbance of each well was measured at a wavelength of 620 nm by microtitre plate reader (Bio-Rad laboratories Inc., India) and compared with a control. Autoclaved water and 'BRN capped Au-NPs' were used as a negative control for each experiment. The procedure was repeated for the pure levofloxacin alone too.

## Results

### Bioconjugation of gold nanoparticles to levofloxacin and drug loading efficiency of levofloxacin

**Figure 2:**
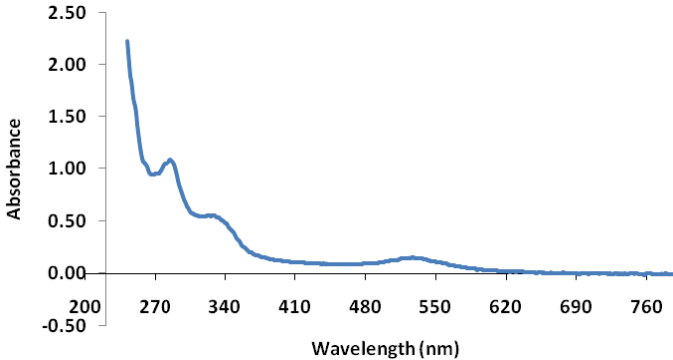
UV-visible absorption spectra of BRN capped gold nanoparticles conjugated with levofloxacin (Au-BRN-LvN-NPs)

The plasmon band was observed for the wine red colloidal gold nanoparticles at 522 nm in the UV-visible spectrum. The colloidal solution containing 'BRN capped Au-NPs' had shown very intense and characteristic pink red color. Two peaks were observed in Au-BRN-LvN-NPs spectrum one at 307 nm and other one at 526 nm during ultraviolet (UV) spectroscopy (Figure 2(Fig. 2)). The drug loading efficiency of LvN was found to be 84.8 ± 2.41 %.

### Measurement of mean particle size and zeta potential of 'BRN capped Au-NPs' and Au-BRN-LvN-NPs

**Figure 3:**
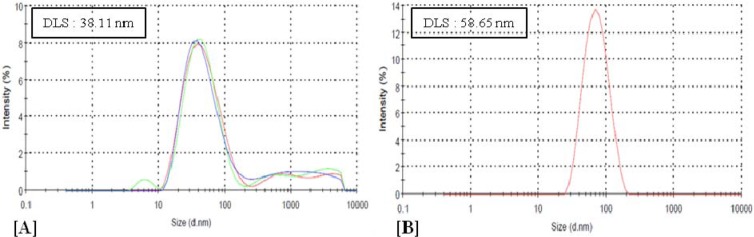
Dynamic light scattering (DLS) analysis of [A] BRN capped gold nanoparticles (BRN capped Au-NPs), and [B] BRN capped gold nanoparticles conjugated with levofloxacin (Au-BRN-LvN-NPs)

**Figure 4:**
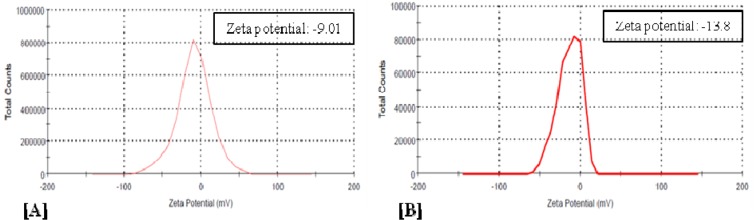
Zetasizer analysis of [A] BRN capped gold nanoparticles conjugated with levofloxacin (Au-BRN-LvN-NPs), and [B] BRN capped gold nanoparticles (BRN capped Au-NPs).

The diameter of Au-BRN-LvN-NPs was found to be 58.65 ± 2 nm while that of its unconjugated form 'BRN capped Au-NPs' as 38.11 ± 2 nm (Figure 3(Fig. 3)). The zeta potential of Au-BRN-LvN-NPs was found to be -9.01 mV and that of its unconjugated form 'BRN capped Au-NPs' -13.8 mV (Figure 4(Fig. 4)).

### Transmission electron microscopy of 'BRN capped Au-NPs' and Au-BRN-LvN-NPs

**Figure 5:**
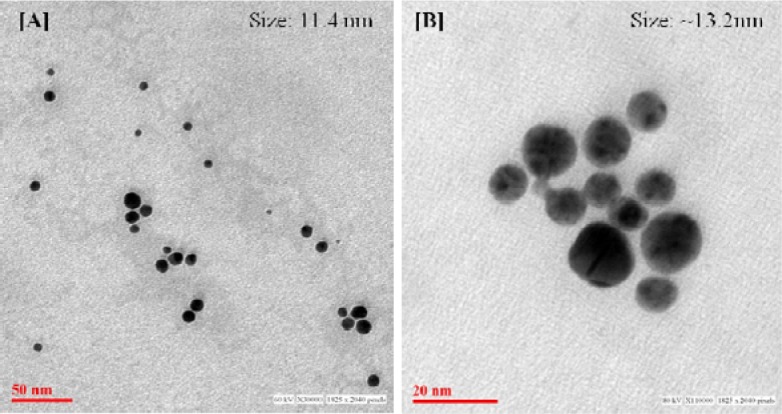
Transmission electron microscopic (TEM) images of [A] BRN capped gold nanoparticles (BRN capped Au-NPs), and [B] BRN capped gold nanoparticles conjugated with levofloxacin (Au-BRN-LvN-NPs)

Surface morphological study of 'BRN capped Au-NPs' and Au-BRN-LvN-NPs showed the formation of spherical particles with the particle size of 11.4 nm of 'BRN capped Au-NPs'. The particle size of Au-BRN-LvN-NPs was found to be ~13.2 nm and it retained the spherical shape (Figure 5(Fig. 5)).

### Fourier transform infrared spectroscopy of 'BRN capped Au-NPs' and Au-BRN-LvN-NPs

**Figure 6:**
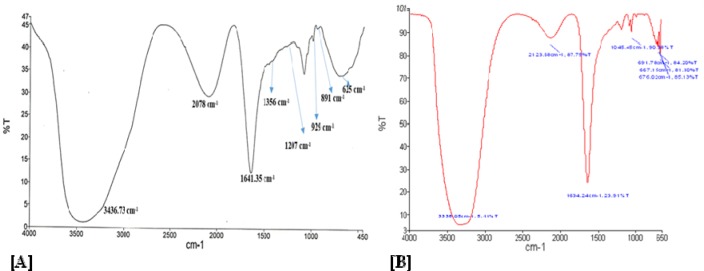
Fourier transform infrared (FTIR) spectra of [A] Pure levofloxacin, and [B] BRN capped gold nanoparticles conjugated with levofloxacin (Au-BRN-LvN-NPs)

FTIR spectrum of 'BRN capped Au-NPs' is shown in Figure 6(A)(Fig. 6) which has showed the characteristic C-N stretch vibration frequencies of monoalkyl guanidinium assigned to the observed IR bands at 1641, 1425-1256 and 1100 cm^-1^. The band at 1760-1670 cm^-1^ (s) showed the presence of C = O groups (amides at ~1640cm^-1^). FTIR spectrum of Au-BRN-LvN-NPs is shown in Figure 6(B)(Fig. 6) which has shown the presence of peak at 1634.24 cm^-1^ due to the presence of C = O (str) of the amide I linkage and the presence of peak at 3338cm^-1^ due OH stretching vibrations.

### Evaluation of antibacterial efficacy of bioconjugated levofloxacin over free levofloxacin

**Figure 7:**
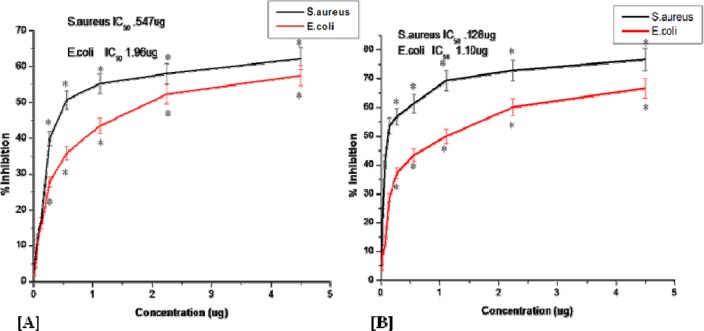
Plot of percent inhibition of bacterial strains versus different concentrations of [A] pure levofloxacin, and [B] BRN capped gold nanoparticles conjugated with levofloxacin (Au-BRN-LvN-NPs)

Percent inhibition of the bacteria was increasing with increasing doses of the pure levofloxacin and Au-BRN-LvN-NPs. The MICs of Au-BRN-LvN-NPs against *S. aureus* (NCIM No. 2079) and *E. coli* (NCIM No. 2065) were found to be 0.128 µg/ml and 1.10 µg/ml respectively, whereas MICs of the pure levofloxacin against *S. aureus* and *E. coli* were found to be 0.547 µg/ml and 1.96 µg/ml respectively (Figure 7(Fig. 7)).

## Discussion

The plasmon band was observed for the wine red colloidal Au-NPs at 522 nm in the UV-visible spectrum which is characteristic of Au-NPs (Liao et al., 2006[17]). The colloidal solution containing 'BRN capped Au-NPs' had shown very intense and characteristic pink red color confirming the formation of Au-NPs. However, two peaks were observed during UV-Visible spectral analysis of Au-BRN-LvN-NPs one at 307 nm due to aromatic acid transitions of LvN and other one at 526 nm due to red shift. This red shift in the plasmon band suggests different dielectric environment which confers conjugation of LvN with 'BRN capped Au-NPs'. The amount of bioconjugated LvN (Au-BRN-LvN-NPs) was found to be 84.8 ± 2.41 % indicating that the LvN was efficiently loaded on synthesized BRN capped Au-NPs by using EDC as the activator.

DLS provides hydrodynamic diameter which includes inorganic core and the thin electric dipole layer of the solvent that adheres to the surface of the nanoparticles (Akhtar et al., 2014[1]). The diameter of Au-BRN-LvN-NPs was found to be 58.65 ± 2 nm while that of its unconjugated form 'BRN capped Au-NPs' 38.11 ± 2 nm proving that bioconjugation of LvN has taken place to form Au-BRN-LvN-NPs. Zeta potential gives an indication of the stability of a colloidal system against agglomeration. The zeta potential of Au-BRN-LvN-NPs was found to be -9.01 mV which is well in range to prevent agglomeration as compared to its unconjugated form 'BRN capped Au-NPs' which was found to be -13.8 mV supporting the absence of nanoparticle aggregation after conjugation. This change in zeta potential may be ascribed to the decrease in number of carboxylic groups which are involved in conjugation of LvN.

During surface morphological study by TEM, the particle size of Au-BRN-LvN-NPs was found to be ~13.2 nm while that of 'BRN capped Au-NPs' as 11.4 nm. It is in coherence with the DLS and further corroborates bionconjugation of LvN with 'BRN capped Au-NPs' and supports the absence of aggregation of nanoparticles after bioconjugation.

From the results of the study, it was found that the percent inhibition of the bacteria *S. aureus* and *E. coli* were increasing with increasing doses of pure levofloxacin and Au-BRN-LvN-NPs suggesting their antibacterial activity against *S. aureus* and *E. coli* in dose dependent manner. The functionalized nanoparticles showed superior antibacterial activity compared to pure LvN at the similar concentration. From the results of the study, it is also clear that there was reduction in the IC_50 _values from 0.547 µg/ml and 1.96 µg/ml of pure LvN to 0.128 µg/ml and 1.10 µg/ml of Au-BRN-LvN-NPs against *S. aureus* and *E. coli* respectively which may be attributed to disruptions in the cell wall and membrane structures of bacteria by it leading to loss of cellular integrity and finally cell death. The IC_50 _value for Au-BRN-LvN-NPs was less against *S. aureus* as compared to that of* E. coli* suggesting that Au-BRN-LvN-NPs are more effective against *S. aureus* bacterium. On the other hand, Au-BRN-LvN-NPs are also able to generate reactive oxygen species which may play a synergistic role in killing bacteria (Nadeau et al., 2008[19]). The probable reasons for superior antibacterial activity of Au-BRN-LvN-NPs may be its superior stability and transport of a huge number of LvN molecules into a highly localized area at the site of particle-bacterium contact (Burygin et al., 2009[4]).

## Conclusion

The results suggest that bromelain capped gold nanoparticles can be used as effective carriers for levofloxacin molecules. The bioconjugated bromelain capped gold nanoparticles exhibited superior antibacterial activity against both Gram negative and Gram positive bacteria compared to pure levofloxacin which may be due to its superior stability and transport of a huge number of LvN molecules into a highly localized area at the site of particle-bacterium contact.

## Acknowledgements

All the authors are highly thankful to the honorable Vice-Chancellor, Integral University, Lucknow, Uttar Pradesh for providing all the necessary facilities related to the present research work. The authors also would like to extend their sincere appreciation to King Saud University, Deanship of Scientific Research, College of Science, Research Centre for its supporting of this research.

## Conflict of interest

The authors declare no conflict of interest.

## Figures and Tables

**Figure 1:**
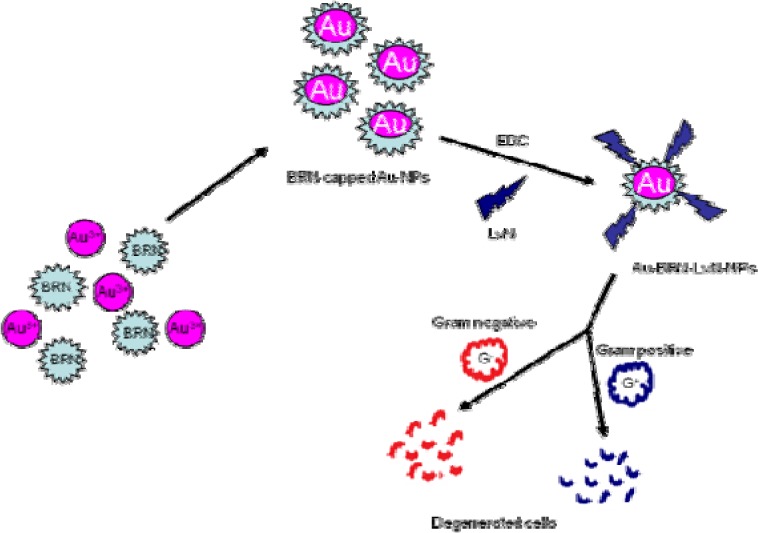
Graphical abstract
